# Diverse modes of galacto-specific carbohydrate recognition by a family 31 glycoside hydrolase from *Clostridium perfringens*

**DOI:** 10.1371/journal.pone.0171606

**Published:** 2017-02-03

**Authors:** Julie M. Grondin, Da Duan, Alyssa C. Kirlin, Kento T. Abe, Seth Chitayat, Holly L. Spencer, Craig Spencer, Alisha Campigotto, Scott Houliston, Cheryl H. Arrowsmith, John S. Allingham, Alisdair B. Boraston, Steven P. Smith

**Affiliations:** 1 Department of Biomedical and Molecular Sciences, Queen’s University, Kingston, Ontario, Canada; 2 Lethbridge Research Centre, Agriculture and Agri-Food Canada, Lethbridge, Alberta, Canada; 3 Department of Biochemistry and Microbiology, University of Victoria, Victoria, British Columbia, Canada; 4 Princess Margaret Cancer Centre and Department of Medical Biophysics, University of Toronto, Toronto, Ontario, Canada; Universiteit Gent, BELGIUM

## Abstract

*Clostridium perfringens* is a commensal member of the human gut microbiome and an opportunistic pathogen whose genome encodes a suite of putative large, multi-modular carbohydrate-active enzymes that appears to play a role in the interaction of the bacterium with mucin-based carbohydrates. Among the most complex of these is an enzyme that contains a presumed catalytic module belonging to glycoside hydrolase family 31 (GH31). This large enzyme, which based on its possession of a GH31 module is a predicted α-glucosidase, contains a variety of non-catalytic ancillary modules, including three CBM32 modules that to date have not been characterized. NMR-based experiments demonstrated a preference of each module for galacto-configured sugars, including the ability of all three CBM32s to recognize the common mucin monosaccharide GalNAc. X-ray crystal structures of the *Cp*GH31 CBM32s, both in apo form and bound to GalNAc, revealed the finely-tuned molecular strategies employed by these sequentially variable CBM32s in coordinating a common ligand. The data highlight that sequence similarities to previously characterized CBMs alone are insufficient for identifying the molecular mechanism of ligand binding by individual CBMs. Furthermore, the overlapping ligand binding profiles of the three CBMs provide a fail-safe mechanism for the recognition of GalNAc among the dense eukaryotic carbohydrate networks of the colonic mucosa. These findings expand our understanding of ligand targeting by large, multi-modular carbohydrate-active enzymes, and offer unique insights into of the expanding ligand-binding preferences and binding site topologies observed in CBM32s.

## Introduction

The human distal gut microbiota is one of the most densely populated microbial ecosystems in Nature, and is home to an elaborate community of bacterial species that live in an intricate, symbiotic relationship with the host [1, 2]. Within this environment, commensal microbes play a critical role in the regular turnover of the protective mucosal layer of the gut, which comprises mucin glycoproteins [3–6]. Several bacteria involved in this process produce and secrete a diverse suite of carbohydrate-active enzymes (CAZymes), particularly glycoside hydrolases [7], suggesting that the recognition, modification, and degradation of the mucin glycans are important for their lifestyle. Enhanced glycan degradation capabilities also enable opportunistic bacterial pathogens to deplete the protective mucosal lining, allowing virulence-associated toxins access to the gut epithelial cell layer [5, 8–10].

One such example is *Clostridium perfringens*, which is a member of the gut microbiota as well as an opportunistic pathogen frequently associated with gastrointestinal infections [11, 12]. The genomes of commensal and infectious *C*. *perfringens* strains contain up to 56 open reading frames coding for glycoside hydrolases of varying known and predicted catalytic activities, of which 13 appear to be secreted into the extracellular milieu [13]. These enzymes are particularly notable for their sizes (i.e., 93–216 kDa) and extensive modularity [14]. In addition to their respective catalytic modules these enzymes comprise differing numbers and types of ancillary modules, which confer a wide variety of complementary functions that mediate simultaneous adherence to target glycans, hydrolysis of target glycans, and the formation of multi-enzyme complexes [14–16].

The most common ancillary module found in secreted *C*. *perfringens* glycoside hydrolases is the carbohydrate-binding module (CBM). CBMs have traditionally been associated with plant cell wall-degrading enzymes, where they function to localize the parent enzymes to appropriate polysaccharide substrates [14, 17, 18]. However, CBMs are increasingly being identified in enzymes involved in the degradation of complex eukaryotic glycans [14]. The array of functions for CBMs in these enzymes, which are often associated with virulence, remain to be fully characterized but in some cases involve bacterial adherence to host glycans [19]. The most highly represented CBM family in *C*. *perfringens* glycoside hydrolases is CBM family 32 (CBM32), which are often found in multiple copies within the parent enzyme [14, 20]. Consistent with these enzymes posited to target the glycan component of mucin, *C*. *perfringens*-derived CBM32s have been shown to display specificities for a diverse set of carbohydrates, including galactose, *N*-acetylgalactosamine (GalNAc), *N*-acetylglucosamine (GlcNAc), *N*-acetyllactosamine (LacNAc; β-D-galactose-1,4-D-*N*-acetylglucosamine), *N*-acetyl-β-D-glucosamine-α-1,4-D-galactose (GlcNAc-α-1,4-Gal), and type II blood group antigen H-trisaccharide [20–24].

**Fig 1:**
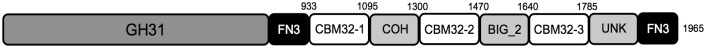
Modular architecture of *Cp*GH31. This enzyme comprises an N-terminal family 31 glycoside hydrolase catalytic module (GH31; dark grey), two fibronectin-type III modules (FN3; black), three family 32 carbohydrate-binding modules (CBM32; white), a putative cohesin module (COH; grey), a bacterial immunoglobulin-like 2 module (BIG_2; grey) and a module of unknown function (UNK; grey). The amino acid borders of the CBM32s are indicated accordingly.

As part of the secreted *C*. *perfringens* CAZyme arsenal, the bacterium possesses an open reading frame coding for a 220-kDa family 31 glycoside hydrolase (locus tag CPF_1301 in strain ATCC 13124) with predicted α-glucosidase activity, which will be hereafter referred to as *Cp*GH31 [13]. In addition to the N-terminal glycoside hydrolase family 31 catalytic module, *Cp*GH31 comprises two fibronectin-type III modules, three CBM32s, a putative cohesin module, and a bacterial immunoglobulin-like 2 module (Fig 1) [25]. Towards understanding the ability of *C*. *perfringens* CAZymes to non-catalytically recognize carbohydrates and to further our understanding of CBM structure-function relationships, we pursued the characterization of the three *Cp*GH31 CBMs by nuclear magnetic resonance (NMR) spectroscopy and X-ray crystallography. Though the putative CBMs, referred to as CBM32-1, CBM32-2, CBM32-3, themselves are not closely related at the amino acid sequence level, a primary structure comparison indicates that they all belong to CBM family 32, an observation that is supported by their overall similar structures as determined by X-ray crystallography. All three *Cp*GH31 CBMs bound the apolar face of GalNAc, while CBM32-1 and CBM32-3 also bound galactose. Integration of NMR-based chemical shift mapping studies with the X-ray crystal structures of the three *Cp*GH31 CBM32s provide insight into the molecular details of how these structurally related CBMs bind the same ligand, GalNAc, but via unique mechanisms.

## Materials and methods

### Cloning, recombinant protein expression, and purification

Gene fragments encoding *Cp*GH31 CBM32-1, CBM32-2, and CBM32-3 were amplified by PCR from *Clostridium perfringens* ATCC 13124 genomic DNA (Sigma; locus tag CPF_1301). Forward and reverse oligonucleotide primers were designed and used for amplification (see Table 1). Gene fragments encoding CBM32-1 (nucleotides 2803–3285), CBM32-2 (nucleotides 3898–4410) and CBM32-3 (nucleotides 4918–5355) were PCR amplified, cloned into pCR8/GW/TOPO plasmids (Invitrogen), and transferred into Champion pET300/NT-DEST Gateway vectors using LR Clonase II (Invitrogen). An 11-amino acid fragment originating from the TOPO vector was transferred into the final vector; however we believe that this in no way impacts the structure and function of the protein. The resulting gene products contained a non-cleavable N-terminal hexa-histidine tag, the small fragment of the TOPO vector, followed by the respective *Cp*GH31 CBM32 sequence (CBM32-1: residues 933–1095; CBM32-2: residues 1300–1470; CBM32-3 residues 1640–1785). A *Cp*GH31 CBM32-2 gene fragment encoding residues 1323–1470 of CPF_1301 was also PCR amplified and subcloned into the *Nde*I and *Xho*I sites of a pET28a expression vector encoding a cleavable hexa-histidine tag. The fidelity of each of the constructs was verified by DNA sequencing. The resultant *Cp*GH31 CBM32-expressing plasmids were transformed into *Escherichia coli* strain BL21 (DE3).

**Table 1:** Oligonucleotide primers used for cloning of the *Cp*GH31 CBM32s.

Primer	Sequence
CBM32-1 (FWD)	5’-GTAAATAAGGATGGACATTC-3’
CBM32-1 (REV)	5’-TCACTTATAAGGTCTAAACTCTC-3’
CBM32-2 long (FWD)	5’-CTGGTTCCGCGTGGATCCGCTAAAATTATTGATTT AGAAAGTG-3’
CBM32-2 long (REV)	5’-CCGCTCGAGCTTCTCATAAAATTCAATTTC-3’
CBM32-2 short (FWD)	5’-GGAATTCCATATGAACATTACTGTAAGTGGTG-3’
CBM32-2 short (REV)	5’-CCGCTCGAGTTATTCACTCTTCTCATAAAATTC-3’
CBM32-3 (FWD)	5’-GATTCTAGCAAGTTAGAAGC-3’
CBM32-3 (REV)	5’-TCATTTTCCGTAGAATACTAATTC-3’

The recombinant unlabeled and ^13^C/^15^N-labeled *Cp*GH31 CBM32 constructs were expressed and purified as N-terminal hexahistidine fusion proteins similar to that previously described for *Cp*GH84A CBM32-1 [26]. The generation of seleno-methionine labeled *Cp*GH31 CBM32-2 comprising residues 1323–1470 involved growth on M9 SeMet high-yield media prepared according to instructions of the manufacturer (Shanghai Medicilon Inc.) at 37°C to an optical density at 600 nm of 1.2, following by addition of seleno-methionine and an inhibitory media cocktail (Shanghai Medicilon Inc.), induction of protein expression by addition of IPTG, and growth at 20°C overnight. Purification of the seleno-methionine labeled CBM32-2 construct involved Ni-NTA affinity chromatography, dialysis against 25 mM Tris-HCl pH 7.5, 50 mM NaCl, cleavage of the N-terminal hexahistidine tag via incubation with thrombin (10 units/mg protein; Sigma) overnight at room temperature, and size exclusion chromatography with a S75 Superdex column (GE Healthcare). In all cases, fractions containing purified proteins were pooled and concentrated, and purity assessed by SDS-PAGE to > 95%.

### NMR spectroscopy

Multidimensional heteronuclear NMR spectra were acquired on Varian INOVA 600 MHz and Bruker 800 MHz spectrometers each equipped with cryoprobes for *Cp*GH31 CBM32-1 (303K). ^1^H, ^13^C, ^15^N backbone and side chain resonance assignments of uniformly ^13^C/^15^N-labeled *Cp*GH31 CBM32-1 (1.3 mM in 25 mM Tris-HCl, pH 6.9, 50 mM NaCl, 90% H_2_O/10% D_2_O) were completed using the following datasets: 2D ^1^H-^15^N HSQC, HNCACB, CBCA(CO)HN, HNCO, HNCACO, and ^15^N-edited NOESY-HSQC (100 ms mixing time). All proton chemical shifts were referenced to DSS. NMR spectra were processed and analyzed using NMRPipe [27] and NMRViewJ [28]. Of the 156 assignable backbone resonances from a total 163 residues in CBM32-1 (excluding Pro955, Pro960, Pro977, Pro999, Pro1053, Pro1068 and Pro1093) 96% of the backbone ^1^H, ^13^C and ^15^N chemical shift resonances were identified.

### NMR-based CBM-carbohydrate titrations

Protein samples comprising 100 μM ^15^N-labeled CBM32-1, 184 μM ^15^N-labeled CBM32-2, and 100 μM ^15^N-labeled CBM32-3 in 25 mM Tris-HCl pH 6.9, 50 mM NaCl and 10% D_2_O had galactose, glucose, GalNAc, GlcNAc, GlcN, and LacNAc added to a final concentration of 8 mM and assessed by 2D ^1^H-^15^N HSQC spectra recorded on a Varian Inova 600 MHz spectrometer at 303K. For quantitative GalNAc titrations, incremental additions of the carbohydrate resulting in total concentrations of 250 μM, 500 μM, 1 mM, 2 mM, 3 mM, 4 mM, 5 mM, 6 mM, 7 mM and 8 mM were added to 500 μM ^15^N-labeled CBM32-1, 410 μM ^15^N-labeled CBM32-2 and 500 μM ^15^N-labeled CBM32-3. Following processing and analysis of the spectra as described above, titration analysis was conducted using CcpNmr Analysis [29]. Non-linear fits were individually applied to protein (A) peaks displaying significantly perturbed backbone amide resonances in the presence of the ligand (B), resulting in the protein-ligand complex (AB) using the quadratic equation:

$$A\left(B+x-\sqrt{{\left(B+x\right)}^{2}-4x}\right)$$

where, *A* = (Δδ_bound_-Δδ_free_)/2 and *B* = 1 + *K*_*d*_/([A]_free_+[AB]), and *x* = ([B]+[AB])/([A]+[AB]). The reported average dissociation constant (*K*_*d*_) values and corresponding standard deviations were calculated from the resulting dissociation constants for the significantly perturbed backbone amide resonances.

### Saturation transfer diffusion (STD) studies

STD NMR spectra were collected on samples dissolved 25 mM phosphate buffer pD 6.9, 50 mM NaCl, 100% D_2_O at 298K on a Bruker 500 MHz spectrometer equipped with a 1.7 mm TCI probe. Spectra on samples containing 100 μM CBM32-1 or 100 μM CBM32-2 were acquired in the absence of and presence of 8 mM GalNAc. Spectra of 250 μM CBM32-3 in the absence and presence of 50 mM GalNAc were acquired. E-Burp2 shaped pulses were employed for saturation of protein resonances with bandwidths of 300 Hz for a period of 2.5 s. Similar ligand STD signals were obtained when protein saturation pulses were centered at -0.5ppm for CBM32-1 and CBM32-2 and 7.5 ppm for CBM32-3. Off-resonance pulses were centered at 35 ppm. 512 transients were collected for each sample. Residual HDO suppression was achieved using excitation sculpting. Processing of the difference spectrum was with exponential line broadening using an LB of 0.1 Hz.

### Crystallization, data collection, and structure determination

Crystallization studies were performed at 291 K or 298 K using the hanging drop vapour diffusion method. CBM32-1 at 11 mg/ml crystallized in 32% PEG 4000, 100 mM Tris-HCl pH 8.0, 225 mM MgCl_2_. A complex of seleno-methionine labeled CBM32-2 with GalNAc was crystallized using a protein concentration of 7.5 mg/ml in 25% PEG 3350, 0.1 M BisTris pH 6.5, 5% glycerol, 10 mM GalNAc. Apo-CBM32-3 at 13 mg/ml was crystallized in 20% PEG 1000, 100 mM HEPES pH 7.5, 50 mM NaCl. CBM32-3:galactose and CBM32-3:GalNAc complex crystals were obtained by first mixing the 10 mg/ml protein sample in 20 mM galactose or 10 mM GalNAc for 1 h, followed by incubation with an equal volume of 1.6 M ammonium citrate. All CBM32-1 and CBM32-3 crystals were cryoprotected using the crystallization solution supplemented with 25% ethylene glycol prior to X-ray diffraction data collection. The CBM32-2:GalNAc complex crystals were cryoprotected in 20% glycerol.

Diffraction data were collected at 100 K at the Advanced Photon Source (Argonne National Laboratory) beamline QM/CA-CAT 23-ID-B, the Canadian Light Source beamline CMCF-BM 08B1-1, the National Synchrotron Light Source (Brookhaven National Laboratory) beamline X6A, and the Stanford Synchrotron Radiation Lightsource (SLAC National Accelerator Laboratory) beamline 14–1.

Reflection data for apo-CBM32-1, apo-CBM32-3, and the CBM32-3:galactose complex were integrated and scaled using HKL-2000 [30], while data for the CBM32-2:GalNAc and CBM32-3:GalNAc complexes were integrated using MOSFLM [31] and scaled using Aimless [32]. The apo-CBM32-1 and apo-CBM32-3 structures were determined by molecular replacement using PHENIX AutoMR [33] with the *Cp*GH84C CBM32 [22] (PDB accession code: 2J1A) as a search model. The refined apo-CBM32-3 model was subsequently used as a search model for molecular replacement-based determination of the CBM32-3:galactose and CBM32-3:GalNAc complex structures. The structure of the CBM32-2:GalNAc complex was determined by single wavelength anomalous dispersion, whereby automated experimental phasing was performed using SHELX [34] and automated model building was done using ARP/wARP [35]. Final models of all five structures were obtained using successive rounds of manual model building in Coot [36] and automated refinement using PHENIX [37] or REFMAC [38]. Model validation was performed using SFCHECK [39] and PROCHECK [40].

### Accession numbers

Structure coordinates reside in the Protein Data Bank (PDB) under accession code 4LPL (apo-CBM32-1), 4UAP (CBM32-2:GalNAc complex), 4LQR (apo-CBM32-3), 4LKS (CBM32-3:galactose complex), and 4P5Y (CBM32-3:GalNAc complex).

## Results and discussion

### Carbohydrate binding preference of the *Cp*GH31 CBM32 modules

Sequence comparison of the three putative CBM32s within the modular architecture of *Cp*GH31 indicated that they share low identity to one another (17–24%). When compared to previously characterized *C*. *perfringens* CBM32s, *Cp*GH31 CBM32-3 showed near-perfect conservation of the canonical galactose-binding residues in the sole CBM32 of *Cp*GH84C and CBM32-5 of *Cp*GH89 [20, 22]. In contrast, clear conservation of residues involved in coordinating canonical galacto- or gluco-configured sugars was not observed in *Cp*GH31 CBM32-1 and CBM32-2, so the sugar-binding specificities of these modules could not be predicted with confidence by sequence comparison alone.

**Fig 2:**
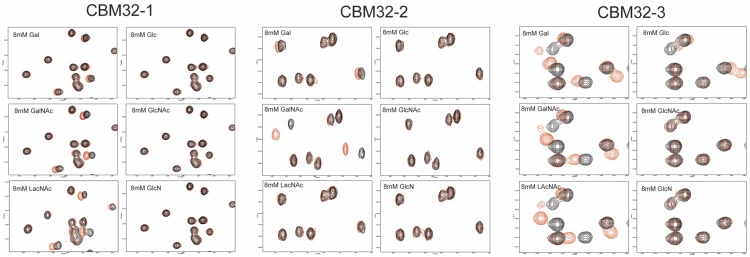
Carbohydrate binding preferences of the *Cp*GH31 CBM32 modules. Regions for the two-dimensional ^1^H-^15^N HSQC spectra of 100 μM CBM32-1, 184 μM CBM32-2, and 100 μM CBM32-3 at pH 6.9 in the absence (black) and presence (red) of 8 mM galactose (Gal), glucose (Glc), N-acetylgalactosamine (GalNAc), N-acetylglucosamine (GlcNAc), N-acetyllactosamine (LacNAc) or N-glucosamine (GlcN).

To assess the specificities of the three *Cp*GH31 CBM32s, binding to an array of carbohydrates, including glucose, GlcNAc, glucosamine (GlcN), galactose, GalNAc, and LacNAc, was monitored by ^1^H-^15^N HSQC NMR experiments (Fig 2). Addition of glucose, GlcNAc or GlcN did not produce observable chemical shift changes in the *Cp*GH31 CBM32-1 spectrum. Rather, residue-specific chemical shift perturbations were observed upon addition of GalNAc and LacNAc. *Cp*GH31 CBM32-3 also displayed a preference for the galacto-configured sugars, as revealed by chemical shift perturbations upon addition of galactose, GalNAc, and LacNAc, an observation consistent with the sequence-based prediction. In contrast, CBM32-2 displayed a clear binding specificity for GalNAc. Subsequent quantitative analysis of NMR-based titrations performed with the three *Cp*GH31 CBM32s using GalNAc as a ligand resulted in dissociation constants (*K*_d_) of 6 ± 2 mM, 0.9 ± 0.4 mM, and 0.9 ± 0.3 mM for the *Cp*GH31 CBM32-1:GalNAc, CBM32-2:GalNAc, and CBM32-3:GalNAc interactions, respectively; values consistent with those binding affinities previously reported for CBM32:sugar interactions [17, 20–24].

### General structural features of the *Cp*GH31 CBM32 modules

The structural basis of the preference for galacto-configured sugars displayed by the three *Cp*GH31 CBM32 modules was investigated by determining the X-ray crystal structures of apo-CBM32-1, a CBM32-2:GalNAc complex, and CBM32-3 in apo-, and in Gal- and GalNAc-bound forms. The structural statistics are summarized in S1 Table.

**Fig 3:**
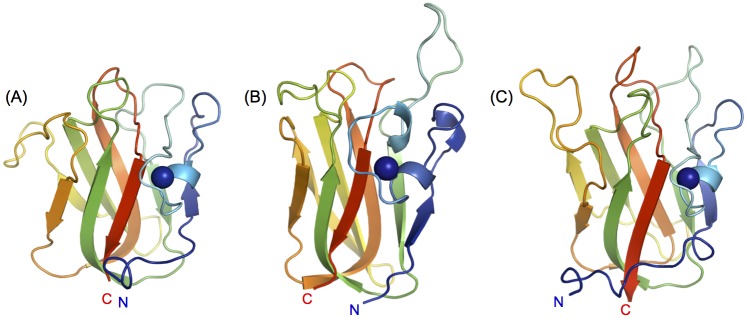
Structures of the *Cp*GH31 CBM32 modules. Cartoon backbone representations of (A) apo-CBM32-1, (B) CBM32-2 in complex with GalNAc, and (C) apo-CBM32-3 determined by X-ray crystallography at 1.35 Å, 2.0 Å and 1.58 Å, respectively. The bound calcium ion is shown in each structure as a blue sphere.

Each *Cp*GH31 CBM32 structure comprised the β-sandwich topology characteristic of this CBM family with a short α-helix and a calcium ion capping one side of each module. Loop regions of variable length extend from the apex of each CBM32, from which a subset forms their respective sugar-binding sites (Fig 3A–3C). The lengths and relative positions of these loops vary between the three CBM32s, consistent with the high sequence divergence in these regions of the primary sequence. Alignment of the core regions of each of the three *Cp*GH31 CBM32s (comprising the β-sandwich) revealed that CBM32-1 and CBM32-3 adopt an overall similar fold (backbone r.m.s.d. of 0.94 Å) while the core structure of CBM32-2 deviates from that of CBM32-1 and CBM32-3 (backbone r.m.s.d. of 2.37 Å and 4.23 Å, respectively).

### Molecular determinants of GalNAc coordination by the *Cp*GH31 CBM32 modules

Analysis of the X-ray crystal structures and complementary NMR spectroscopic studies allowed for the identification of the binding determinants for galacto-configured sugars by the three *Cp*GH31 CBMs, including the displayed GalNAc specificity by CBM32-2.

**Fig 4:**
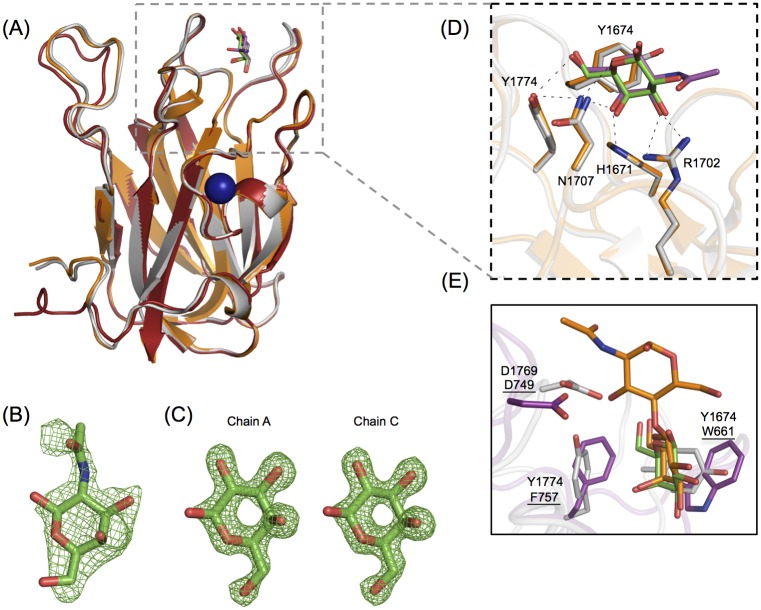
*Cp*GH31 CBM32-3 recognizes GalNAc with the same set of residues employed by canonical galactose-binding CBM32s. (A) Backbone cartoon structure overlay of the X-ray crystal structures of CBM32-3 in the apo-form (red) and in complex with galactose (grey) and GalNAc (orange), determined to 1.58 Å, 1.48 Å and 2.50 Å, respectively. Galactose is shown in green, and GalNAc in magenta. The Ca^2+^ ion observed in all three structures is depicted as a blue sphere. (B) Electron density of GalNAc bound to the single peptide chain of the CBM32-3:GalNAc structure, with the *F*_*obs*_*-F*_*calc*_ electron density map contoured to 2.5 σ. (C) Galactose bound to monomer chains A and C in the CBM32-3:galactose complex structure, with the *F*_*obs*_*-F*_*calc*_ electron density map contoured to 3.0 σ. (D) Expanded view of structural overlay of the ligand-coordinating residues from the galactose-bound (grey) and GalNAc-bound (orange) structures from (A). The same set of residues is involved in binding both ligands via hydrogen bonds (shown as black dashes). (E) A structural overlay of *Cp*GH31 CBM32-3 (grey) bound to galactose (green, coordinating residues denoted) with *Cp*GH84C CBM32 (magenta, coordinating residues underlined) bound to LacNAc (orange, PDB code 2J1E; [22]).

The molecular determinants of galacto-specific sugar recognition displayed by *Cp*GH31 CBM32-3 were revealed by the X-ray crystal structures of this protein module in its apo-, galactose-bound, and GalNAc-bound states, which were determined to 1.58 Å, 1.48 Å and 2.50 Å resolution, respectively (Fig 4). Alignment of the three CBM32-3 structures revealed minimal sugar—induced structural changes (backbone r.m.s.d < 0.22 Å) and only subtle differences in B-factors associated with the variable loops were also observed (Fig 4A). These findings are suggestive of a preconfigured conformation for carbohydrate recognition with limited dynamic properties, which is a structural feature previously observed for other CBMs [24, 41, 42]. The galactose and GalNAc residues were well-ordered in the crystal structures of the CBM32-3:Gal and CBM32-3:GalNAc complexes, respectively, and provided clear electron density, allowing for monosaccharide modeling into each structure (Fig 4B and 4C).

The galactose and GalNAc coordinating amino acid residues of CBM32-3 were conserved, and included Tyr1674, His1671, Arg1702, Asn1707 and Tyr1774. The b-face of both sugars formed CH-π interactions with Tyr1674, while a secondary aromatic residue, Tyr1774, whose side chain is perpendicular to that of Tyr1674, mediated additional hydrophobic interactions with the C6 group of each sugar (Fig 4D). The use of tyrosine as the main aromatic stacking residue has been previously observed in *C*. *perfringens* CBM32s [20], a role usually filled by a conserved tryptophan residue [20–24]. Galactose and GalNAc binding was further stabilized through a series of polar contacts formed between the side chains of His1671, Arg1702 and Asn1707 and the C2-C3-C4-C5 edge of each sugar. Specifically, hydrogen bonds were observed between Asn1707 and the O4 hydroxyl and endocyclic oxygen, His1671 and the O4 hydroxyl, and Arg1702 and the O3 and O4 hydroxyl and 2-acetamido groups (Fig 4D). The orientation of galactose and GalNAc, and the respective contacts being made with the CBM were consistent with the STD NMR data, in which strong STD signals for the apolar α- and β-H3, α- and β-H4, α/β-H6, and acetyl group protons were observed (see panel A in S1 Fig).

This structural information also provides a basis with which to predict the interactions CBM32-3 may form with LacNAc, an interaction detected in NMR titrations (Fig 2). The C6 of galactose is well-positioned to interact with the hydrophobic region formed by the adjacent side chains of Tyr1774 and Tyr1674, leaving the C1 of the sugar directed toward the solvent, which would allow for the binding of the β1-4-coordinated dissacharide LacNAc at this position (Fig 4E). Previous studies on the LacNAc-bound structure of the canonical galactose-binding CBM32 from *Cp*GH84C identified an aspartate (Asp749) in a loop adjacent to the binding site that was well-positioned to make hydrogen-bonding interactions with the GlcNAc moiety of LacNAc [22]. Although this residue is not conserved in *Cp*GH31 CBM32-3, the side chain of Asp1769 is similarly positioned in an adjacent loop and could form hydrogen bond contact LacNAc.

**Fig 5:**
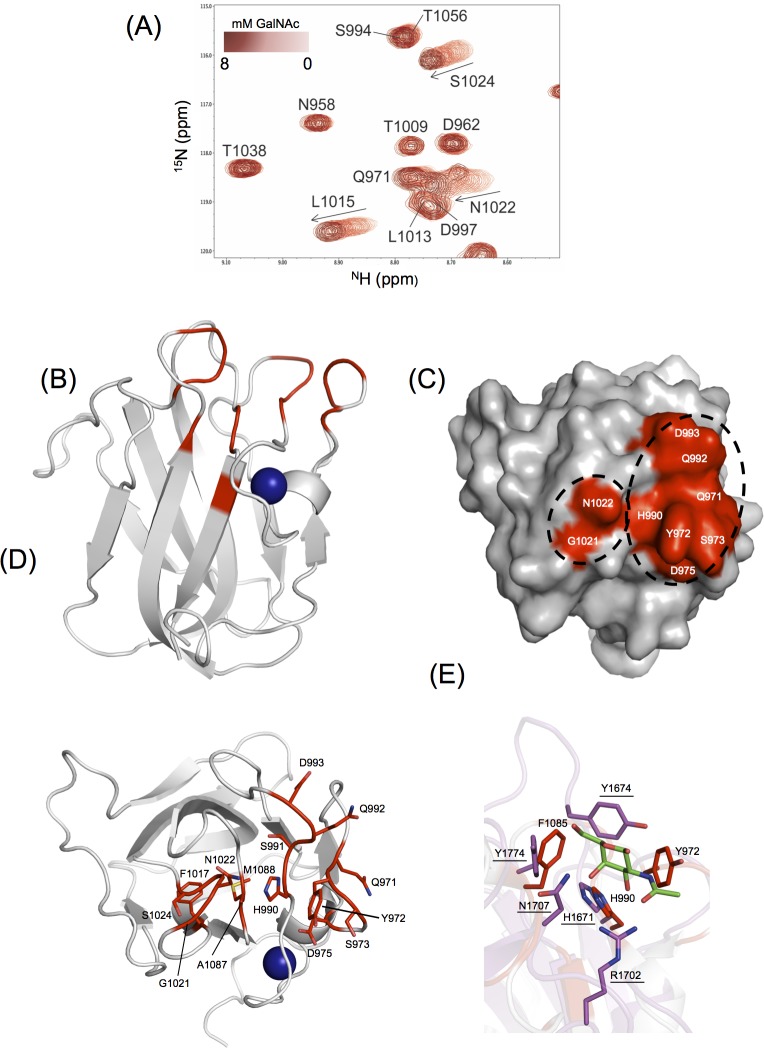
Identification of the *Cp*GH31 CBM32-1 GalNAc binding site. (A) Region of overlaid ^1^H-^15^N HSQC spectra of 500 μM CBM32-1 with increased amounts of GalNAc. (B) Backbone cartoon representation of the X-ray crystal structure of apo-CBM32-1 with residues whose backbone amide resonances were significantly perturbed (>1 standard deviation above the mean chemical shift change) in the presence of GalNAc shown in red. The calcium ion is shown as a blue sphere. (C) Surface representation of apo-CBM32-1 on which those significantly perturbed backbone amide resonances are displayed in red and identified in white as single-letter amino acid code. (D) Amino acid residues of CBM32-1 (shown as sticks and identified by single-letter code and position in *Cp*GH31 sequence) whose backbone amide resonances display significant GalNAc-induced chemical shift changes are coloured red on a backbone cartoon representation of apo-CBM32-1. (E) Comparison of the GalNAc binding sites of *Cp*GH31 CBM32-1 (grey, select residues denoted) and *Cp*GH31 CBM32-3 (magenta, residues underlined) reveal structural conservation of Phe1985 and His990 with Tyr1774 and His1671, respectively. Tyr972 and Tyr1674 are located in different variable loop regions but are similarly positioned in the binding site.

Multi-dimensional heteronuclear NMR experiments were used to map the GalNAc binding site on the apo-CBM32-1 structure, determined to 1.35 Å resolution, as co-crystallization and soaking attempts of *Cp*GH31 CBM32-1 with galacto-configured sugars were unsuccessful (Fig 5). A subset of backbone amide resonances that corresponded to residues primarily grouped to four distinct regions of the CBM32-1 backbone were significantly perturbed upon addition of GalNAc, including Gln971, Tyr972, Ser973, Asp975; His990, Ser991, Gln992, Asp993, Leu1015, Gly1021, Asn1022, Gly1023, Ser1024; Ala1087, Met1088, and Glu1090 (Fig 5A). When mapped onto the apo-CBM32-1 structure, the four regions formed a contiguous site localized to variable loops located at the apex of the CBM, comprising an aromatic residue (Tyr972) and several polar residues (Gln971, Asp975, Ser973, His990, Gln992, Asp993, and Asn1022), consistent with previously identified CBM32 carbohydrate-bindings sites (Fig 5B–5D).

Saturation transfer difference (STD) NMR spectroscopic analysis revealed strong STD signals for the apolar α- and β-H3, α- and β-H4, α/β-H6, and acetyl group protons whereas the STD signals of the H1 protons were attenuated (see panel B in S1 Fig). These observations suggest that the C1 edge of GalNAc is solvent-exposed and thus accessible for extension of a glycan chain at this position, while the remainder of the sugar would interact with CBM32-1 via hydrophobic and hydrogen bonding contacts. The topology of the binding site and the STD NMR data are also consistent with the more general ability of CBM32-1 to specifically recognize galacto-configured sugars.

Two aromatic residues with side chains perpendicular to one another are also a common feature of the sugar-binding site of CBM32s [43]. Heteronuclear NMR titration experiments identified one aromatic residue significantly perturbed in the presence of GalNAc (Tyr972), as well as a second (Phe1085), which experienced backbone chemical shift perturbations greater than the mean value but less than one standard deviation above the mean (Fig 5E). As the crystal structure of CBM32-1 lacks a sugar ligand, the exact role of each of these aromatic residues in coordinating GalNAc is uncertain. However, the secondary aromatic residue (Tyr1774) and binding site histidine (His1671) of the CBM32-3:GalNAc complex described above are structurally conserved with corresponding residues in the binding site of CBM32-1 (Phe1085 and His990, respectively) (Fig 5E). The other tyrosine residues implicated in both binding sites (Tyr972 in CBM32-1 and Tyr1674 in CBM32-3) are oriented similarly despite being located on different variable loops. These similarities hint at a similar mode of GalNAc coordination between CBM32-1 and CBM32-3.

**Fig 6:**
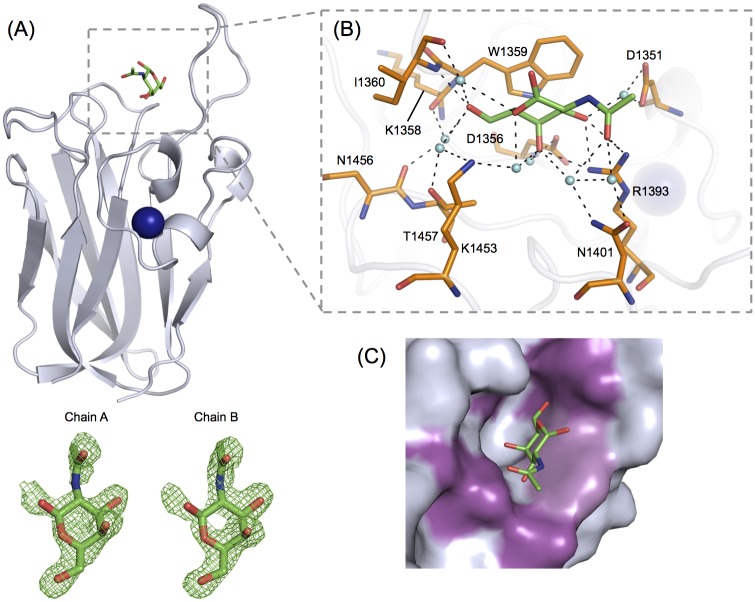
GalNAc binding determinants of *Cp*GH31 CBM32-2. (A) Backbone cartoon representation of CBM32-2 (grey) in complex with GalNAc (green), solved to a resolution of 2.00 Å. The associated calcium ion is depicted as a blue sphere. *F*_*obs*_*-F*_*calc*_ electron density maps of GalNAc (green) bound to peptide chains A and B of the CBM32-2:GalNAc structure are shown in green mesh and contoured to 3.0 σ. (B) GalNAc (green) is bound to CBM32-2 by a several aromatic and polar residues (orange) via direct and water-mediated hydrogen bonds. Associated water molecules are shown as cyan spheres and hydrogen bonds are depicted by dashed lines. The stacking interaction is mediated by Trp1359. (C) The shallow GalNAc-specific binding site of CBM32-2 (shown in grey) accommodates the O4 hydroxyl group of the ligand in an axial position only. The sugar associates with the side chain of Trp1359 (light purple) and forms numerous hydrogen-bonding interactions (magenta) that target the O6 hydroxyl and 2-acetamido groups on either end of the sugar.

A structural rationale for the observed GalNAc specificity displayed by *Cp*GH31 CBM32-2 was provided by the X-ray crystal structure of the CBM32-2:GalNAc complex determined to 2.0 Å (Fig 6). The GalNAc was well-ordered in the crystal structure and provided sufficiently clear electron density for modelling the monosaccharide into the binding site of *Cp*GH31 CBM32-2 (Fig 6A). GalNAc was coordinated by a subset of residues located in the variable loop region at the apex of CBM32-2 (Fig 6A and 6B). The side chain of Trp1359 formed the main aromatic-based CH-π interaction between the b-face of GalNAc and the protein surface. Notably, the binding site lacked additional sugar-coordinating aromatic residues, which is an unusual feature among clostridial CBM32s that has only been previously observed in the non-canonical galactose-binding *Cp*GH84A CBM32-1 [24]. To compensate for the lack of supporting non-polar contacts, several polar residues were observed to interact with the C3-C4-C5-C6 edge of GalNAc via an extensive network of direct and water-mediated hydrogen bonds: the side chains of Arg1393 and Asp1351 with the O3 hydroxyl, the side chains of Arg1393, Asp1356 and Lys1453 with the O4 hydroxyl, the side chain of Thr1457 and backbones of Lys1358 and Ile1360 with the O6 hydroxyl, the backbone of Ile1360 and side chain of Lys1453 with the endocyclic oxygen, and the side chains of Asp1351, Arg1393 and Asn1401 with the 2-acetamido group (Fig 6B). The O1 hydroxyl was directed towards the solvent and did not make any contacts with the protein surface. The coordination of the C3-C4-C5-C6 edge of the binding site was in accordance with the STD-NMR signals observed for the GalNAc H3, H4 and H6 protons, as well as the attenuation of signal of the H1 protons (see panel C in S1 Fig).

With respect to the observed GalNAc specificity by CBM32-2, the shallow binding surface accommodated the hydroxyl O4 group of the sugar in the axial position only (Fig 6C). As such, the topology of the binding site cannot accommodate gluco-configured ligands such as glucose, GlcNAc, or GlcN. In order to selectively bind GalNAc over other galacto-configured ligands (i.e., galactose and LacNAc), polar residues in the binding site were positioned to form water-mediated hydrogen bonding interactions to the 2-acetamido group in addition to the coordination of the O6 hydroxyl groups of the sugar (Fig 6B). This novel bilateral hydrogen-bonding network has not been previously observed for a CBM-sugar interaction. Rather, surface residues of the CBM are typically positioned to coordinate only one end of monosaccharide ligands. Since the hydrogen bonds involving the extended 2-acetamido group of GalNAc and the side chains of Asp1351, Arg1393 and Asn1401 were observed to be water-mediated, the distance between these side chains and the shorter O2 hydroxyl group of galactose or the galactose moiety of LacNAc may be too great to allow for these ligands to adhere to the protein surface.

### Comparison of *Cp*GH31 CBM32s binding sites

**Fig 7:**
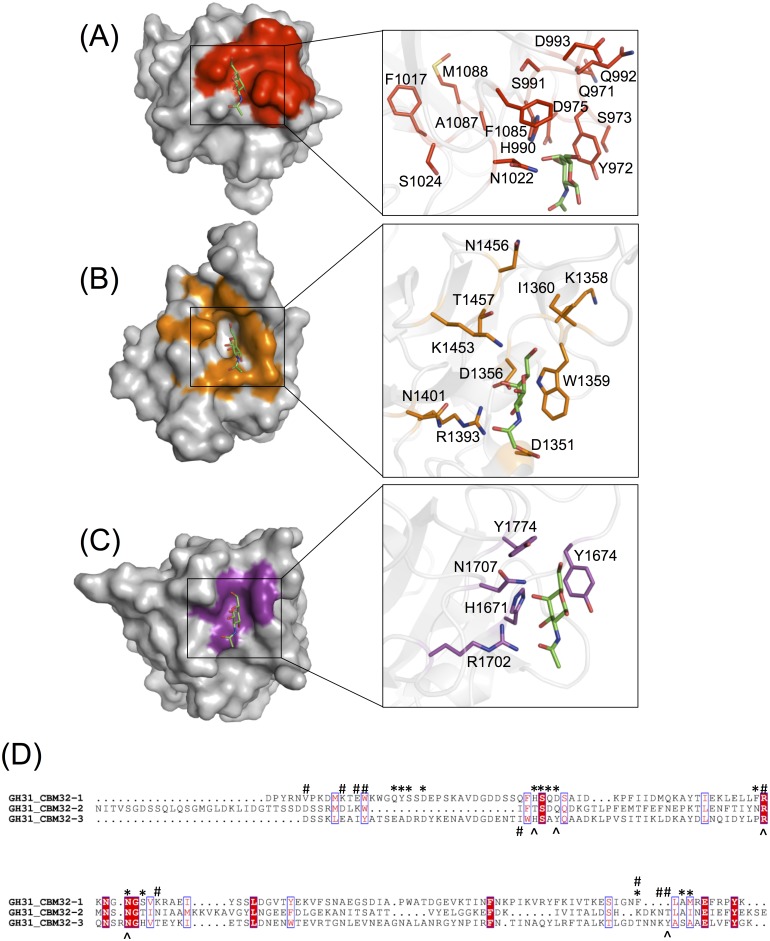
Comparison of the GalNAc binding sites of the *Cp*GH31 CBM32s. The variable loop regions of (A) CBM32-1 (red), (B) CBM32-2 (orange), and (C) CBM32-3 (violet) contain a similar complement of residues involved in the recognition of GalNAc (green), including one or more aromatic residues. The structural conservation of key residues in the variable loop regions of CBM32-1 and CBM32-3 (His990, Phe1085, Tyr972 and His1671, Tyr1774, Tyr1674, respectively) allowed for GalNAc to be modeled into the binding site of CBM32-1. Specifically, *Cp*GH31 CBM32-3:GalNAc was identified as the top structural homologue of CBM32-1 using the DALI server (Z-score of 18.1; backbone r.s.m.d. of 1.9 Å), and the two structures were superimposed in order to position GalNAc into the binding site of CBM32-1. The binding site of CBM32-2 is unique as these residues are not conserved, but rather replaced by an extensive suite of residues involved in the coordination of GalNAc via hydrogen bonding. Residues are represented by their single-letter amino acid code. (D) Amino acid sequence alignment of the three *Cp*GH31 modules. Positions comprising conserved amino acid residues are identified by white single-letter code and highlighted in red while positions displaying amino acid residues of similar physicochemical properties are identified by red-single letter code. An asterisk denotes amino acid residues of *Cp*GH31 CBM32-1 implicated in GalNAc recognition by NMR titrations while those coordinating GalNAc in the *Cp*GH31 CBM32-2 and CBM32-3 are identified by pound and ampersand symbols, respectively. The sequence alignment was created using CLUSTAL OMEGA [45, 46] and displayed using ESPript [47].

Overall, the modes of GalNAc recognition displayed by the three *Cp*GH31 CBM32s involved a similar composition of amino acid residues, which clustered to the same three regions on the respective CBM32 structures (Fig 7A–7D). A comprehensive phylogenetic analysis of over 200 individual CBM32s from CAZymes across several bacterial species highlight the pronounced sequential diversity in the CBM32s from *C*. *perfringens*, and defines distinct modes of sequence variability among CBM32s found within and between individual enzymes, termed homogeneous and heterogeneous clustering [44]. In homogeneous clustering, two or more CBM32s from the same enzyme display the highest degree of sequence similarity with one another, which is thought to result in similar, overlapping sugar binding profiles. In contrast, heterogeneous clustering refers to CBM32s from one enzyme that display the highest percentage sequence identity to CBM32s from a different enzyme grouping and is suggested to translate into different sugar binding profiles for each CBM32 in a given multi-modular enzyme. According to this phylogenetic analysis the *Cp*GH31 CBM32s exhibit heterogeneous clustering, as they display a higher degree of sequence similarity with CBM32s from various bacterial sources than amongst themselves. Interestingly, the overlapping sugar binding panels of the *Cp*GH31 CBM32s contradicts those predicted by heterogeneous clustering, whereby sequential divergence would translate to varied ligand binding profiles. This striking discrepancy between clustering patterns and sugar binding preferences underpins the insufficiency of attempting to classify CBM32 binding patterns based solely on primary sequence.

The sequential divergence of the three *Cp*GH31 CBM32s is underscored by the distinct lack of GalNAc binding residue conservation in the primary sequence of the three modules (Fig 7). Common among all three sites is the inclusion of one or more aromatic residues, positioned to coordinate the b-face of the pyranose ring, or to orient the C1 toward the solvent in order to discern terminal sugar moieties from longer glycan chains, as well as several polar residues, including conserved histidine residues His990 and His1671 in CBM32-1 and CBM32-3, respectively. In the case of CBM32-2, the hydrogen-bonding network common among CBM32 binding sites is adapted to fill the position vacated by the absence of the secondary aromatic residue (Fig 7B).

**Fig 8:**
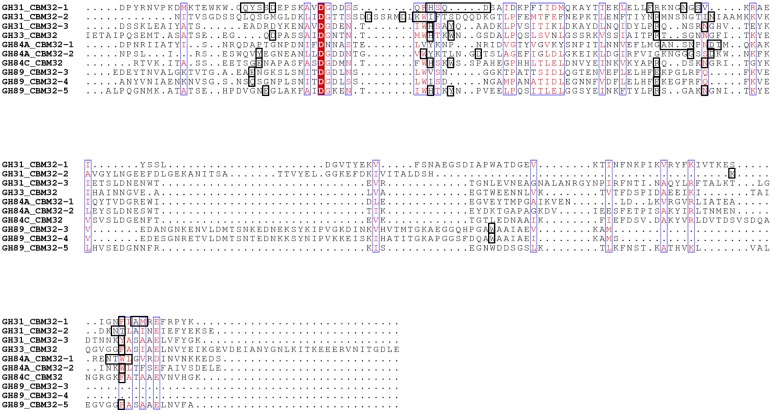
Amino acid sequence comparison of GalNAc-binding *C*. *perfringens* CBM32s. Sequence alignment of the three *Cp*GH31 CBM32 modules with other functionally characterized CBM32 modules from the following family 33, family 84, and family 89 glycoside hydrolases with specificity for galacto- or gluco-configured sugars: *Cp*GH33 CBM32, galacto-configured sugar specificity [21]; *Cp*GH84A CBM32-1, galacto-configured sugar specificity [24]; *Cp*GH84A CBM32-2, GlcNAc specificity [23]; *Cp*GH84C CBM32, galacto-configured sugar specificity [22]; *Cp*GH89 CBM32-3 and CBM32-4, GlcNAc-α-1,4-Gal specificity [20]; *Cp*GH89 CBM32-5, galacto-configured sugar specificity [20]. Positions comprising conserved amino acid residues are identified by white single-letter code and highlighted in red while positions displaying amino acid residues of similar physicochemical properties are identified by red-single letter code. Sugar-coordinating amino acid residues in each CBM32 seequence are identified by black boxes. The sequence alignment was created using CLUSTAL OMEGA [45, 46] and displayed using ESPript [47].

Functional subtleties such as these have also been observed in the analyses of the binding site compositions of other clostridial CBM32s (Fig 8) [20–24], as well as in those of CBMs from families 35, 51 and 71. Similar to CBM32s, these latter families comprise exo-type CBMs involved in the coordination of the terminal galactose moiety of a variety of carbohydrate motifs [43, 48–52]. The growing library of CBM32-carbohydrate complex structures and corresponding identification of critical ligand-coordinating residues underscores, with the exception of the canonical galactose coordination model displayed by a subset of clostridial CBM32s, a general lack of strict conservation of residues involved in binding specific sugars, despite occupying similar positions along the primary amino acid sequence (Fig 8). The redundancy in sugar binding profiles despite a paucity of sequence conservation among the three CBM32s of *Cp*GH31, in conjunction with the functional diversification of binding site signatures, is a notable example of the growing range of finely-tuned structural variations available in this sequentially diverse CBM family.

Together, the canonical galactose-based recognition of GalNAc by CBM32-3, the binding of GalNAc with an allowance for LacNAc and galactose by CBM32-1, and the strict preference of CBM32-2 for GalNAc suggest that this ligand is the core unit required for substrate recognition by *Cp*GH31, an observation consistent with prevalence in the *O*-glycans of the colonic mucosa in humans and animals [10, 53]. *O*-glycosylation involves the attachment of a GalNAc moiety at its C6 carbon to a serine or threonine residue on the associated protein component of the glycoprotein, which is central to the four core structures identified in human colonic mucins [10, 53, 54]. Thus, the ability of a carbohydrate-modifying enzyme to recognize this central monosaccharide is critical. The preferential binding of GalNAc by the *Cp*GH31 CBM32s suggests that this enzyme may target GalNAc-rich regions of mucin for enzymatic degradation.

CBMs have long been known to play a key role in the enhancement of activity of their parent enzymes, which include secreted bacterial CAZymes and virulence factors. A particularly prevalent and diverse CBM family found in CAZymes of the gut microbiome is the CBM32 family. An increasing number of unique features are being uncovered for CBM32s from *C*. *perfringens* CAZymes. What was first thought to be a CBM family based on the canonical recognition of galactose, is one that has rapidly evolved into a relatively unpredictable and diverse group of CBMs with the ability to recognize a variety of sugar ligands using a varied set of molecular features [44]. The CBMs from *Cp*GH31 exhibit both canonical galactose-binding properties, as well as unique modes of GalNAc recognition that are not predictable based on primary structure comparisons.

An interesting trend is emerging from the characterization of multimodular *C*. *perfringens* CAZymes, where the enzymatic activity of the catalytic module rarely matches the binding preferences of the resident CBM32 modules [14, 21, 25, 51]. Examples to date include a family 89 glycoside hydrolase from *C*. *perfringens*, which displays α-*N*-acetylglucosaminidase activity and while it does contain two CBM32 modules with affinity for GlcNAc-α-1,4-Gal, also includes a canonical galactose-binding CBM32, two non-functional CBM32s and one CBM32 of unknown function [20], and a family 84 glycoside hydrolase (*Cp*GH84C) that possesses exo-β-D-*N*-acetylglucosaminidase activity yet contains a single canonical galactose-binding CBM32 [22]. SAXS studies on *Cp*GH84C provide the first clue in our understanding of CBM contribution to enzyme function when considering a complex glycan substrate. The heterogeneous sugar composition of a complex glycan, such as those present on the mucosal surface, necessitates *C*. *perfringens* CAZymes be able adhere to the substrate via a sugar moiety that does not necessarily correspond to that recognized by the catalytic module but that might be in close proximity. The active site of the catalytic module and binding site of the CBM in *Cp*GH84C are spatially coordinated within the full-length enzyme to optimize the binding and hydrolysis of complex glycans [25]. *Cp*GH31 is predicted to have α-glucosidase activity, and as shown here the three resident CBM32s display specificity for galacto-configured sugars. These latter observations for *Cp*GH31 are consistent with the general trend of multimodular *C*. *perfringens* CAZymes, and appear to represent an adaptation of these enzymes to interact with and degrade structurally complex mucin glycans. Unique and diverse glycan-binding properties of resident CBMs that complement but do not overlap with the specificity of the catalytic modules in these enzymes ensure the two modules are not competing for binding the same target glycand and that latter is brought into close proximity of its substrate.

## Supporting information

S1 Fig STD NMR of *Cp*GH31 CBM32:GalNAc interactions. The ^1^H reference spectra (top) and STD NMR spectra (bottom) of (a) 50 mM GalNAc in the presence of 250 μM CBM32-3, and 8 mM GalNAc in the presence of (b) 100 μM CBM32-1, (c) 100 μM *Cp*GH31 CBM32-2.
(PDF)

S1 Table X-ray data collection and refinement statistics for the *Cp*GH31 CBM32s. (PDF)
