# Supplementary material for: Diverse modes of galacto-specific carbohydrate recognition by a family 31 glycoside hydrolase from Clostridium perfringens
Source: PLoS One. 2017 Feb 3;12(2):e0171606. doi: 10.1371/journal.pone.0171606 (PMC5291390; doi:10.1371/journal.pone.0171606)
Supplement: S1 Fig — The 1H reference spectra (top) and STD NMR spectra (bottom) of (a) 50 mM GalNAc in the presence of 250 μM CBM32-3, and 8 mM GalNAc in the presence of (b) 100 μM CBM32-1, (c) 100 μM CpGH31 CBM32-2. (PDF) [file pone.0171606.s001.pdf]

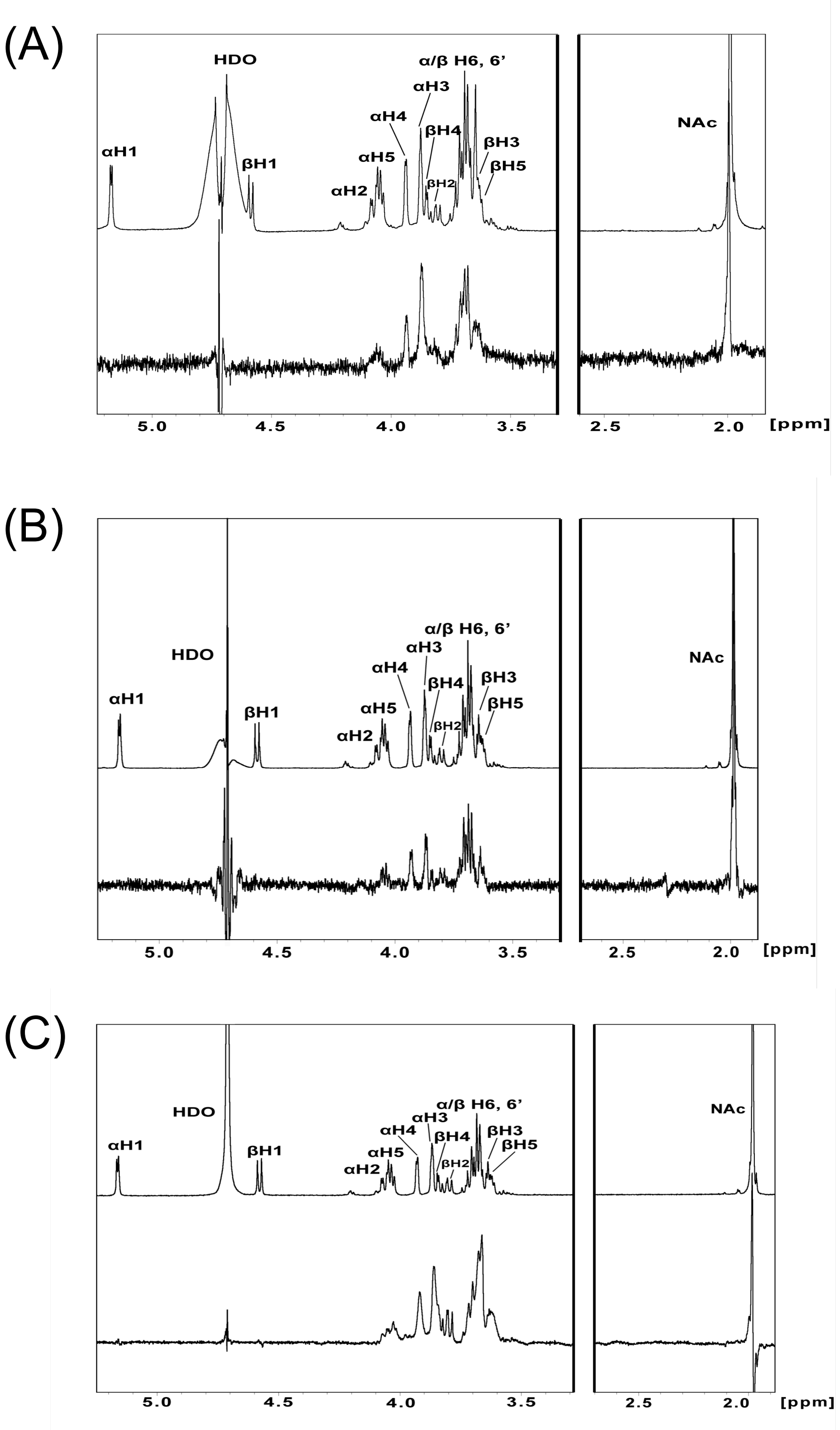

S1 Fig. STD NMR of *Cp*GH31 CBM32:GalNAc interactions. The ^{1}H reference spectra (top) and STD NMR spectra (bottom) of (a) 50 mM GalNAc in the presence of 250 μM CBM32-3, and 8 mM GalNAc in the presence of (b) 100 μM CBM32-1, (c) 100 μM *Cp*GH31 CBM32-2.
