# Supplementary material for: Diverse modes of galacto-specific carbohydrate recognition by a family 31 glycoside hydrolase from Clostridium perfringens
Source: PLoS One. 2017 Feb 3;12(2):e0171606. doi: 10.1371/journal.pone.0171606 (PMC5291390; doi:10.1371/journal.pone.0171606)
Supplement: S1 Table — (PDF) [file pone.0171606.s002.pdf]

S1 Table. X-ray data collection and refinement statistics for the *Cp*GH31 CBM32s.

**Data collection statistics**	**CBM32-1 (apo)**	**CBM32-2 (GalNAc)**	**CBM32-3 (apo)**	**CBM32-3 (galactose)**	**CBM32-3 (GalNAc)**
Wavelength (Å)	1.033	0.979	1.055	0.98	0.98
Beamline	APS 23-ID-B	CLS 08B1-1	NSLS X6A	NSLS X6A	SSRL 14-1
Space group	$C222_1$	$P22_12_1$	$P2_12_12_1$	$P4_32_12$	$P4_32_12$
Resolution range	30.00-1.35 (1.40-1.35)	38.41-2.00 (2.05-2.00)	50.00-1.58 (1.64-1.58)	50.00-1.48 (1.53-1.48)	42.89-2.50 (2.60-2.50)
Cell dimension	55.62, 55.67, 88.70	48.30, 84.75, 86.17	38.76, 52.56, 70.17	62.33, 62.33, 85.35	60.549, 50.549, 83.69
α, β, γ, (°)	90.00, 90.00, 90.00	90.00, 90.00, 90.00	90.00, 90.00, 90.00	90.00, 90.00, 90.00	90.00, 90.00, 90.00
R_{meas}	0.063 (0.45)	0.16 (0.52)	0.042 (0.084)	0.057 (0.38)	0.114 (0.513)
Completeness (%)	98.9 (97.7)	99.9 (100)	98.9 (91.0)	99.9 (99.2)	100.0 (100.0)
<I/σI>	23.8 (4.2)	13.7 (7.8)	34.7 (26.6)	31.5 (6.3)	14.3 (3.8)
Redundancy	5.7 (5.1)	14.0 (14.1)	7.8 (7.6)	8.4 (8.0)	6.0 (6.2)
Total reflections	171557	345760	157870	242452	35305
Unique reflections	30335	24623	20155	28797	5836
**Refinement statistics**					
R_{work} (%)	18.9	19.2	16.1	17.6	17.4
R_{free} (%)	20.7	22.1	19.4	20.2	22.1
r.m.s.d.					
Bond lengths (Å)	0.006	0.020	0.024	0.006	0.007
Bond angles (°)	1.066	1.928	2.248	1.042	1.016
Average B-factors (Å^2)					
Protein atoms chain A	15.471	14.906	8.266	14.436	28.966
Protein atoms chain C	-	16.384	-	-	-
Water molecules	23.924	26.716	20.399	26.655	34.197
Ligand	-	30.178	-	28.518	44.594
Cations	10.335	8.660	9.189	15.400	39.150
Number of atoms					
Protein atoms chain A	1152	1175	1277	1257	1235
Protein atoms chain C	-	1156	-	-	-
Water molecules	114	352	193	229	52
Ligand	-	50	-	24	30
Cations	2	2	2	1	1
Ramachandran statistics					
Most favoured (%)	97.1	98.0	98.1	96.9	96.8
Additional allowed (%)	2.9	2.0	1.9	3.1	3.2
Disallowed (%)	0.0	0.0	0.0	0.0	0.0
Protein Data Bank code	4LPL	4UAP	4LQR	4LKL	4P5Y

*Numbers in brackets refer to values from the highest resolution shell
